# Xylitol Modification of Electrospun Polymer Scaffolds: Impact on Physicochemical and Antibacterial Properties

**DOI:** 10.3390/polym17223024

**Published:** 2025-11-14

**Authors:** Francesco Boschetto, Matteo Zanocco, Kaeko Kamei, Huaizhong Xu, Elia Marin

**Affiliations:** 1 Ceramic Physics Laboratory, Faculty of Materials Science and Engineering, Kyoto Institute of Technology, Sakyo-ku, Matsugasaki, Kyoto 606-8585, Japan; 2 Department Polytechnic of Engineering and Architecture, University of Udine, 33100 Udine, Italy; matteo.zanocco@uniud.it (M.Z.); elia-marin@kit.ac.jp (E.M.); 3 Department of Biomolecular Engineering, Kyoto Institute of Technology, Sakyo-ku, Matsugasaki, Kyoto 606-8585, Japan; kame@kit.ac.jp; 4 Department of Biobased Materials Science, Kyoto Institute of Technology, Sakyo-ku, Matsugasaki, Kyoto 606-8585, Japan; xhz2008@kit.ac.jp; 5 Biomaterials Engineering Laboratory, Faculty of Materials Science and Engineering, Kyoto Institute of Technology, Sakyo-ku, Matsugasaki, Kyoto 606-8585, Japan; 6 Biomedical Research Center, Kyoto Institute of Technology, Sakyo-ku, Matsugasaki, Kyoto 606-8585, Japan

**Keywords:** electrospinning, composites, xylitol, antibacterial

## Abstract

Electrospun fibrous scaffolds based on cellulose acetate (CA), polycaprolactone (PCL), and poly (L-lactic acid) (PLLA) are versatile materials with applications spanning diverse fields, but in their pristine form, they typically lack significant inherent antibacterial properties. To address this limitation and expand their utility, this study explored the incorporation of xylitol, a natural antibacterial sugar alcohol, into these polymer matrices to enhance their physicochemical and antimicrobial properties. Electrospinning was employed to fabricate pristine and xylitol-loaded scaffolds with varying xylitol concentrations. Morphological analysis revealed polymer-dependent changes in fiber diameter and porosity. Mechanical testing assessed the impact of xylitol on tensile properties, while thermal analysis investigated alterations in melting temperature and crystallinity. The antibacterial efficacy against *Staphylococcus aureus* and *Escherichia coli* was evaluated using WST assay and live/dead staining. Notably, xylitol significantly enhanced the antibacterial activity against both bacterial species, with a more pronounced and rapid effect observed against *S. aureus*. The tailored scaffold properties and imparted antimicrobial characteristics highlight the potential of these xylitol-modified electrospun materials: they are easily produced, low-cost, and appropriate for a range of applications (dental applications, filters, masks, wound dressing, and packaging) where preventing bacterial contamination is crucial.

## 1. Introduction

The development of fibrous scaffolds with tailored structural and functional properties has gained considerable attention for a wide range of biomedical and environmental applications, including wound dressings, tissue engineering, air and water filtration, and drug delivery systems [1,2,3,4,5,6,7]. Electrospun fibrous scaffolds have emerged as promising platforms, owing to their high surface area-to-volume ratio, tunable porosity, and morphological similarity to the extracellular matrix (ECM) [8,9]. Among the various fabrication techniques, electrospinning has proven especially versatile for producing ultrafine fibers from both synthetic and natural polymers [10,11]. This method enables the creation of nanofibrous materials with controlled architecture, mechanical properties, and functional surface chemistry, which are key attributes for biomedical and industrial applications. Polycaprolactone (PCL), a semicrystalline aliphatic polyester, is not only well-established in biomedical applications, such as bone regeneration and drug delivery, but is also used in 3D printing, adhesives, and agricultural films due to its biodegradable nature and favorable mechanical properties [10,12,13,14]. Poly(L-lactic acid), PLLA, a more rigid biodegradable polymer derived from renewable resources, has demonstrated utility in tissue engineering scaffolds and resorbable implants, while also serving as a key material in packaging, textiles, and 3D printing [15,16,17,18]. Cellulose acetate (CA), a derivative of natural cellulose, is valued for its excellent film-forming ability and has been used in filtration membranes, biomedical coatings, and sustainable packaging [19,20,21]. However, the inability of these three polymers to inherently inhibit microbial growth limits their effectiveness in scenarios where sterility or resistance to infection is crucial. Many applications, particularly in healthcare and environmental protection, require not only structural and mechanical performance, but also antibacterial or bacteriostatic properties to prevent infection and contamination. To address this limitation, a common strategy involves the development of composite materials by incorporating antimicrobial agents or fillers, either synthetic (e.g., silver nanoparticles, antibiotics) or natural (e.g., chitosan, plant-derived compounds), into the polymer matrix [22,23,24,25,26,27,28,29,30]. These additives can provide multifunctional behavior, enhance not only antibacterial activity but also influence other material properties such as porosity, degradation rate, and surface chemistry. Among natural fillers, sugar alcohols, so-called bio-based polyols, have attracted considerable attention, being commercially important chemicals for green materials [31]. Among these molecules, glycerol is well-known and utilized in many applications, pharmaceuticals, cosmetics, and the food industry, often as a humectant, solvent, and emollient [32]. Particularly, it has been promoted in food packaging where its role as a plasticizer is valued for providing the developed films with the necessary flexibility [33]. Another important sugar alcohol is xylitol, which stands out due to its well-documented antibacterial and anti-adhesive properties [34,35]. Xylitol is renowned especially in dentistry for its remarkable ability to prevent dental caries (tooth decay) and enhance oral health [36]. Unlike traditional sugars, it is not fermentable by oral bacteria, and this characteristic inhibits the acid production that typically leads to enamel demineralization and cavity formation [37]. Instead, bacterial uptake of xylitol leads to a futile metabolic cycle where ATP is consumed but no energy is generated, resulting in growth inhibition. Additionally, xylitol reduces bacterial adhesion by disrupting the synthesis of extracellular polysaccharides and glycocalyx, thereby impairing biofilm formation and colony stability. This dual action—starving bacteria of energy while limiting their ability to establish protective biofilms—confers broad-spectrum antibacterial properties [35,38,39]. Xylitol has been shown to reduce plaque formation and increase saliva production, which helps in the remineralization of tooth enamel and the maintenance of a neutral pH in the oral cavity [36]. And beyond its dental benefits, xylitol has been demonstrated to help in preventing ear infections in children by hindering bacteria from attaching to nasal passages, and to control the growth of skin microbes [40,41,42]. However, unlike glycerol, which has been studied previously in composite development, there are no works already published that describe the possibility of fabricating a new class of electrospun composites of polymers and xylitol presenting antimicrobial properties. Furthermore, compared to other compounds like quaternary ammonium salts (QASs) and silver nanoparticles (AgNPs), xylitol can represent a superior choice as an antibacterial filler primarily because of its selectivity and safety profile. Glycerol, a humectant and plasticizer, lacks significant inherent antimicrobial properties; QASs are broad-spectrum, potentially irritating, and can disrupt beneficial microbiota; and AgNPs raise concerns regarding long-term toxicity and environmental impact. In contrast, xylitol is a non-toxic and natural sugar alcohol that selectively targets and inhibits different bacteria strains by interfering with their metabolism and adhesion, making it significantly safer and more desirable for applications requiring chronic or direct human contact. These effects are particularly relevant in biomedical scaffolds, where xylitol incorporation can significantly reduce bacterial colonization and prolong material functionality. This study aims to investigate the effect of incorporating different concentrations of xylitol into electrospun fibrous scaffolds composed of PCL, PLLA, and CA. A comprehensive characterization of the physical, mechanical, and structural properties of these composites was performed, focusing on how xylitol concentration influences fiber morphology, porosity, thermal and mechanical behavior, and its antimicrobial potential functionality, aiming to create low-cost scaffolds suited for advanced biomedical or industrial applications, even in environments with limited access to medical resources.

## 2. Materials and Methods

### 2.1. Sample Preparation

The three polymeric electrospun solutions were fabricated using different methods:

- CA pellets with an average molecular weight (M_w_) of 30,000 (Sigma Aldrich, St. Louis, MO, USA) were dissolved in acetone (Sigma Aldrich, St. Louis, MO, USA);
- PLLA pellets with a M_w_ of 106,000 (Musashino Chemical Laboratory, Tokyo, Japan) were added to chloroform (FUJIFILM Wako Pure Chemical Corporation, Osaka, Japan);
- PCL pellets with an average molecular weight (M_n_) of 80,000 (Sigma Aldrich, St. Louis, MO, USA) were dissolved in a mixture of chloroform/acetone in a ratio of 3/1.

All the solutions were prepared at a concentration of 20% *w*/*v* and mixed using a magnetic stirrer at room temperature for 24 h, until fully dissolved.

Subsequently, solutions of xylitol at increasing concentrations were prepared by dissolving xylitol powder (FUJIFILM Wako Pure Chemical Corporation, Osaka, Japan) in distilled water. These xylitol solutions were added dropwise to each polymer solution under continuous stirring, maintaining a consistent volume ratio of 9 parts polymer solution to 1 part xylitol solution. The mixtures were then stirred for an additional 24 h at room temperature. This specific volume ratio was calculated to yield three distinct weight ratios of polymer to xylitol in the final electrospinning solutions: 67/33, 50/50, and 33/67. Final solutions were collected in a 1 mL syringe with a 24-gauge metal needle and processed for electrospinning.

Optimal process parameters (voltage, flow rate, and needle-collector distance) were allowed to provide stability of the electrostatic jet during electrospinning. A positive voltage of 10–12 kV was applied to the needle using a high voltage power supply (Model-600F, Pulse Electronic Engineering Co., Ltd., Noda, Japan). The different solutions were extruded through the horizontal-oriented needle using a syringe pump at a flow rate of 0.25 μL/s. A stainless cylindrical steel mandrel (diameter: 2 mm, length: 10 cm) as a collector was placed at a distance of 10 cm from the needle tip. A grounded aluminum sheet was placed at the back of mandrel to induce the fiber collection on the mandrel. The conditions were kept constant across the three polymers to allow for clear comparisons. The final samples were obtained after a deposition under environmental temperature of 25 °C and controlled relative humidity of 30%.

For clarity, sample nomenclature was defined as follows: CA, PLLA, and PCL refer to cellulose acetate, poly (L-lactic acid), and polycaprolactone, respectively. The suffixes -X1, -X2, and -X3 indicate increasing xylitol concentrations, with -X1 representing the lowest (33% *w*/*w*) and -X3 the highest (67% *w*/*w*).

### 2.2. Morphological, Physical, and Chemical Characterization

A scanning electron microscope (SM-700, JEOL, Tokyo, Japan) was used to acquire high magnification images of the samples before the biological testing. The samples were sputter-coated with approximately 2 nm of platinum and observed at an accelerating voltage of 10 kV. Five images for each group of samples have been collected at different magnifications (500×, 1000×, and 2000×) (*n* = 5). Fiber diameter and porosity have been calculated by analyzing 100 and 150 fibers per group of samples, respectively. Fiber density analysis utilized three images per class, with all data analyzed using ImageJ (version 1.52a, Rasband, W.S., ImageJ, National Institutes of Health, Bethesda, MD, USA).

To detect the presence and distribution of xylitol, Raman imaging was performed using a confocal Laser Raman microscope (RAMANtouch, Nanophoton Co., Ltd., Osaka, Japan). The excitation sources were 532 nm, at a nominal power of 200 mW. In order to prevent samples from burning, the power output was controlled by adjusting a dedicated ND filter. The maps were obtained by using lenses having 20× magnification with numerical apertures ranging from 0.5 to 0.23, and for each image, arrays of 400 points were acquired simultaneously and combined in a bi-dimensional map. The acquisition time was fixed at 10 s for each point. From each maps an average spectrum was obtained and deconvoluted into Gaussian–Lorentzian sub-bands using commercially available software (Labspec5, version 5.58.25, Horiba/Jobin–Yvon, Kyoto, Japan).

Contact angle measurements were conducted to assess surface wettability using a Phoenix 300 apparatus (Kromtek Co., Selangor, Malaysia) operating at room temperature. The droplets were analyzed using ImageJ. For each sample, 5 μL droplets of deionized water were dropped onto the surface of the substrates, and the angle was then measured. The average water contact angle value was obtained by measuring five different locations across the surface for each group of samples (*n* = 5).

### 2.3. Mechanical Tests

Mechanical properties of the different scaffolds were calculated using a tensile tester (STA-1150, ORIENTEC, Tokyo, Japan). Each sample was tested at a 20 mm clamping distance, stretched at a rate of 10 mm/min, at room temperature. The measurements were repeated six times for each class of samples (*n* = 6).

### 2.4. Thermal Characterization

Differential Scanning Calorimetry (DSC) was performed using a DSC-50 thermal analyzer (Shimadzu, Kyoto, Japan). Samples of approximately 2 mg were sealed in aluminum pans and heated up from room temperature to 250 °C at a heating rate of 10 °C/min under a nitrogen flow of 15 mL/min. Analyses were replicated three times/group of samples (*n* = 3), and all the spectra were analyzed using a software provided by the machine vendor to calculate the melting temperature (*T_m_*) and the melting enthalpy (Δ*H_m_*). Subsequently, with a commercial software (Origin, version 9.8.0.200, Originlab Corp., Northampton, MA, USA) the degree of crystallinity (%*C*) was obtaining according to the following equation:

$$\%C=\frac{{\Delta H}_{m}}{{\Delta H}_{m}^{{}^{\circ}}}\times 100$$

where ${\Delta H}_{m}^{{}^{\circ}}$ is the melting enthalpy of completely crystalline CA (58.8 J/g), PCL (139.5 J/g), and PLLA (93 J/g) [43,44,45].

### 2.5. In Vitro Test

Gram-positive bacteria, *Staphylococcus aureus* (*S. aureus*, NBRC 13276) and Gram-negative bacteria, *Escherichia coli* (*E. coli*, E1 NBRC 3972), were cultured using a brain–heart infusion (BHI) liquid medium (Sigma Aldrich, Tokyo, Japan). The initial 1.8 × 10^10^ CFU/mL was subsequently diluted to 1.8 × 10^8^ CFU/mL using a phosphate-buffered saline (PBS, NACALAI TESQUE.INC, Kyoto, Japan) solution to mimic ion blood concentrations. The samples with diameters of 1 cm were sterilized prior to the experiment using a UV sterilizer (254 nm, Phillips, Denver, CO, USA) for 24 h. Then the samples were incubated at 37 °C for 24 and 48 h.

### 2.6. Bacterial Characterization

WST is a well-established technique to measure bacterial metabolism by calorimetric detection. In this experiment, the WST-8 kit (Microbial Viability Assay Kit-WST, Dojindo, Kumamoto, Japan) was used as a calorimetric indicator which releases a water-soluble formazan dye upon reduction in the presence of an electron mediator. The amount of the formazan dye generated is linearly related to the number of living microorganisms. The solution is subjected to microplate readers (EMax, Molecular Devices, Sunnyvale, CA, USA) upon collecting the OD value related to living cells. Three samples of each group were used to calculate the average values (*n* = 3).

After exposure to bacteria at different intervals of time, the bacteria were stained using a *Bacstain* Bacterial Viability Detection Kit-CFDA/PI provided by Dojindo (Laboratories, Kumamoto, Japan), presenting two different solutions to evaluate their conditions: propidium iodide (PI) and 5(6)-carboxyfluorescein diacetate (CFDA). The red color of PI, if entered inside the cells, highlighted dead or injured bacteria, while the CFDA’s green color revealed esterase activity and thus cell membrane integrity in living bacteria. The staining protocol consisted of adding 1 μL of the PI solution and 15 μL of CFDA solution to the samples, and then incubating them for 5 min at 37 °C. After removing the buffer, the cells were analyzed under the fluorescence microscope (BZ-X700; Keyence, Osaka, Japan) at 4× magnification. Three samples of each group were used to calculate the average values (*n* = 3).

### 2.7. Statistical Analysis

The experimental data were analyzed with respect to their statistical meaning by computing their mean value ± one standard deviation. The statistical significance has been addressed by one-way ANOVA for materials characterization analysis (contact angle, thermal and mechanical tests evaluation); for the bacterial tests, two-way ANOVA analysis of variance was used. Statistically significant results (*p* < 0.05) have been marked with an “*”.

## 3. Results

### 3.1. Effect of Xylitol on Fiber Morphology

**Figure 1:**
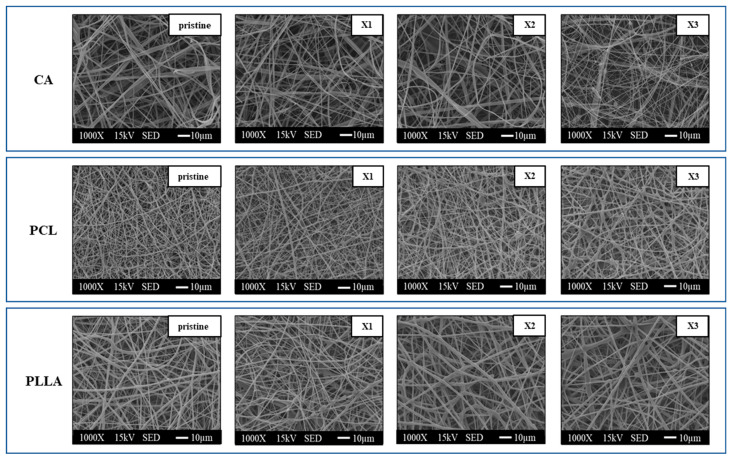
SEM images of the CA, PCL, PLLA, and their fibers loaded with xylitol at increasing concentrations.

Figure 1 reveals the surface morphology of the pristine and xylitol composite fibers of CA, PCL, and PLLA. For CA, the incorporation of low xylitol concentrations (CA-X1, CA-X2) did not visibly alter fiber size or the homogeneous distribution observed in the pristine CA scaffold. However, CA with the highest xylitol content (CA-X3) displayed non-uniform fiber diameters, particularly due to the presence of thinner fibers, and produced a few beads along the fibers. Unlike CA, both PCL and PLLA pristine and low xylitol concentration composites exhibited homogeneous fiber distributions without any noticeable bead formation. PCL showed a change in fiber diameter with increasing xylitol concentration, a trend not evident in PLLA; furthermore, like in the case of CA-X3, beads with different sizes were observed also in PCL-X3 and PLLA-X3.

**Figure 2:**
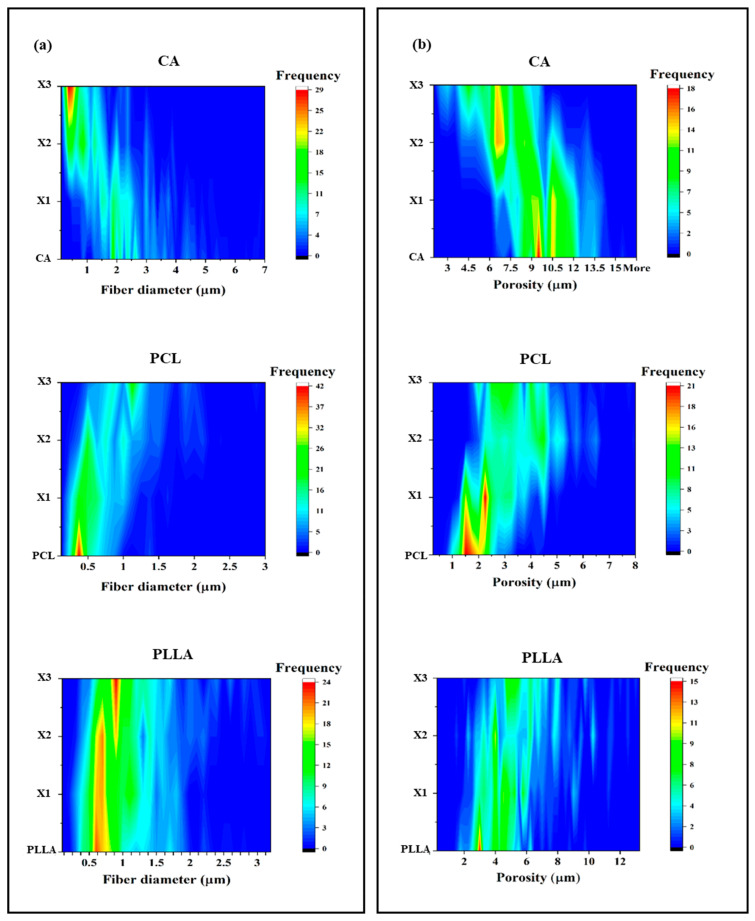
(**a**) Fiber diameter distribution and (**b**) porosity and its frequency, for each group of composites.

Figure 2a shows three plots representing the fiber diameters of the three types of polymeric fibers and their corresponding xylitol composites. The increase in the amount of xylitol brought a clear reduction in the average fiber diameter, going from pure CA fibers (average diameter of 2.92 ± 1.41 µm) to the composites (2.17 ± 1.05, 1.15 ± 0.79, and 0.74 ± 0.57 µm for CA-X1, CA-X2 and CA-X3, respectively). The distribution was less uniform with the incorporation of the xylitol, particularly in the case of CA-X3, where most of the fibers presented a diameter of less than 500 nm. For PCL (central panel of Figure 2a), an opposite trend of fiber diameter distribution occurred, confirming what was previously observed by SEM (Figure 1). PC-X3 scaffold displayed the bigger diameter with an average of 1.09 ± 0.45 µm. For PCL pristine, almost all the fibers analyzed presented a diameter inferior than 1 µm (0.45 ± 0.24 µm), while for the other two composites, the distribution of the fiber diameter was more homogeneous and the average size was bigger than the one of the untreated samples (0.58 ± 0.29 and 0.90 ± 0.48 µm for PC-X1 and PC-X2, respectively). In the case of PLLA (bottom panel of Figure 2a), no significant differences have been detected. The distribution and the average of the fiber diameter did not vary between the pristine (0.94 ± 0.50 µm), PL-X1 (0.89 ± 0.41 µm), and PL-X2 (1.03 ± 0.53 µm). In the case of PL-X3, the distribution of the diameter was more uniform while the average diameter slightly increased (1.11 ± 0.55 µm).

The pore size distribution across CA, PCL, and PLLA composites demonstrates a clear influence of increasing xylitol content on pore size and frequency. In Figure 2b, Pristine CA (top panel) exhibits the highest pore size (average of 10.2 ± 1.7 µm), with dominant peaks around 9.5 µm and 10.5 µm, and a broader distribution between 9 and 13 µm. This indicates that CA inherently forms a more open, porous matrix. As xylitol is added, the structure becomes more compact: the CA-X1 blend shows slightly reduced pore sizes (mostly 8–12 µm, with an average porosity of 9.8 ± 1.8 µm) with still noticeable peaks of frequency at 9.5 and 10.5 µm but at lower intensities. At CA-X2 (7.7 ± 1.9 µm), the distribution becomes bimodal, with two clear peaks around 6.5 µm and 8 µm, suggesting a transitional structure with both medium and small pores. With the highest xylitol content (CA-X3), the matrix is dominated by smaller pores (4–8 µm), with a strong frequency peak at 7 µm, highlighting xylitol’s ability to significantly reduce pore size and increase uniformity (average porosity of 6.07 ± 1.80 µm).

Unlike CA, pristine PCL starts with a very tight pore size distribution (average pore size of 1.75 ± 0.57 µm), heavily centered around 2 µm, indicating a dense and compact microstructure with low permeability (central panel in Figure 2b). The PCL-X1 sample maintains a similar trend but with a slightly broader range with an increase in average pore size to 2.47 ± 0.87 µm, suggesting a partial disruption of the polymer matrix. At the 50:50 ratio (PCL-X2), a more heterogeneous porosity with a higher average (3.9 ± 1.3 µm) emerges, with dual frequency peaks around 2.5 µm and 4–5 µm, pointing to increased structural diversity that could enhance diffusion. The PCL-X3 blend shows a clear shift toward larger pores with a distribution between 4 and 5 µm and an average of 3.3 ± 1.1 µm. Furthermore, it displayed a decreased material density, likely due to xylitol interfering with polymer chain packing.

For PLLA, the pore size pattern is initially consistent and narrow, and the trend of increasing xylitol concentration is similar to the one observed in the PCL composites (bottom panel Figure 2b). Pure PLLA shows a dominant frequency peak around 3.5–4 µm, with most of the pores between 2 and 6 µm, reflecting a dense and uniform structure with average pore size of 3.9 ± 1.4 µm. In PLLA-X1, the pore size range widens (average of 4.8 ± 1.8 µm), extending up to 8 µm, indicating the onset of structural heterogeneity, while at PLLA-X2, the pore distribution becomes broader (approximately 3–10 µm), and the peak shifts to higher pore sizes (with an average increase of 5.56 ± 2.38 µm), likely due to phase separation or solubilization effects. The PLLA-X3 blend exhibits the most diffuse and widespread distribution, with frequencies extending up to 12 µm. Although the highest concentrations remain between 4 and 6 µm, the increased variability suggests a highly porous (average of 6.06 ± 2.38 µm) and less uniform matrix.

**Figure 3:**
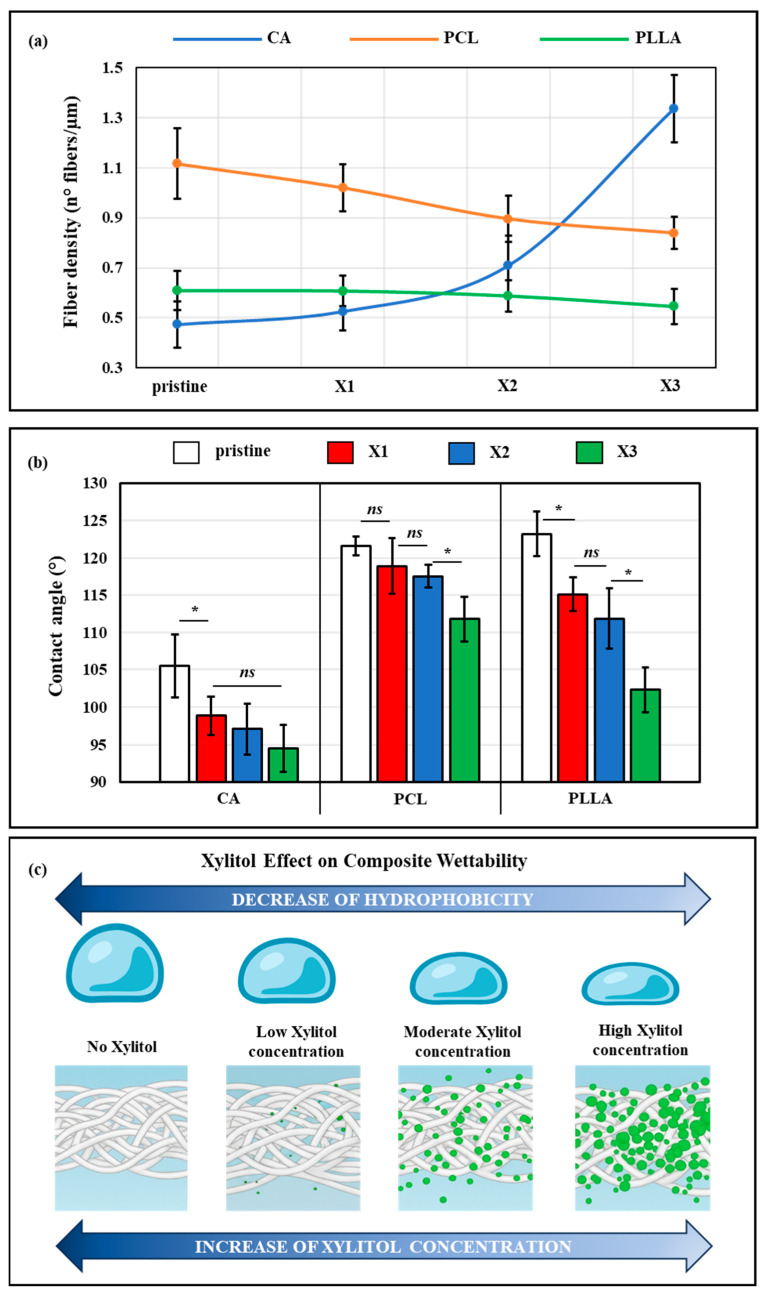
(**a**) Fiber density calculation, (**b**) contact angle for each group of composites, and (**c**) schematic diagram of correlation between xylitol content and wettability (*ns* = non-significant; * = *p* < 0.05).

Figure 3a displayed the fiber density of the samples, expressed as the number of fibers encountered for linear micron of length. PLLA scaffolds showed a constant value of density of the fibers with increasing concentrations of xylitol, while for the PCL samples, this value progressively decreased, ultimately becoming significantly smaller at PC-X3. The CA pristine presented the lowest fiber density of the three classes of polymers. However, at high xylitol concentrations, a marked increase in density was observed, attributed to the reduction in fiber diameter as previously noted.

The contact angle analysis of Figure 3b indicates that the incorporation of xylitol significantly influences the surface wettability of the electrospun scaffolds composed of CA, PLLA, and PCL. Among the pristine polymers, PLLA and PCL displayed the highest hydrophilicity with a contact angle of 123.0 ± 3.0° and 121.6 ± 1.3°, respectively, while CA showed more hydrophilic behavior, with contact angles of 105.5 ± 4.2°. Upon the addition of xylitol, a notable reduction in contact angle was observed for all polymer types, particularly in the case of PLLA where the PLLA-X3 sample showed a value of 102.3 ± 2.7°. Similarly, CA and PCL composites showed gradual decreases in contact angle concurrently to the increase in xylitol concentration, with PCL-X3 and CA-X3 achieving a contact angle of 111.8 ± 3.0° and 94.5 ± 2.7°, respectively, and demonstrating how xylitol effectively enhances the hydrophilic character of the scaffolds. Figure 3c summarizes the results that emerged from the contact angle measurement, showing a clear decrease in hydrophobicity correlated with an increase in xylitol concentration.

### 3.2. Raman Characterization and Xylitol Location

**Figure 4:**
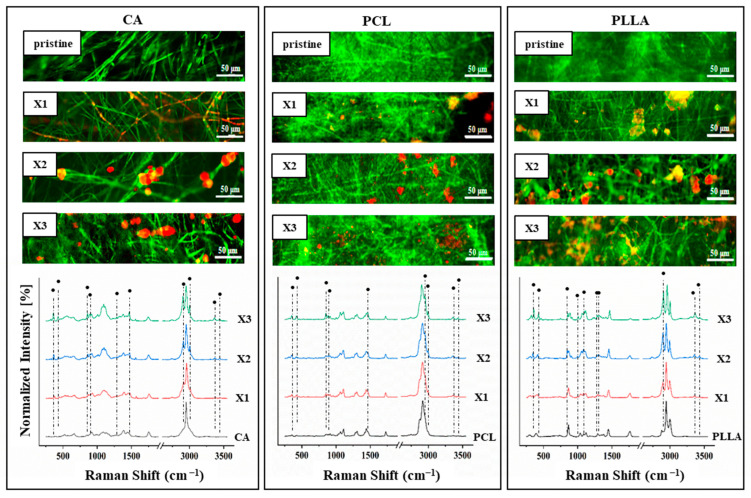
Raman spectra and related images after selecting one band related to the polymer matrix (labeled in green) and the bands at (2953 and 3000 cm^−1^) to detect the xylitol (labeled in red).

Raman maps allow us to observe and characterize the distributed xylitol by monitoring the spectral intensity at specific sugar-related peaks. All the average spectra of the polymer/xylitol composites displayed in Figure 4 are characterized by the presence of a series of bands labeled with a brand and associated with vibrational modes belonging to the sugar.

In particular, different characteristic bands of xylitol are observed in specific spectral positions and related to CC aliphatic chain vibration (357 and 427 cm^−1^), CO stretching (859 and 889 cm^−1^), CC alicyclic and aliphatic chain vibration (1068, 1097 and 1286 cm^−1^), CH_2_ bending (1468 cm^−1^), CH stretching (2953, 3000 cm^−1^), and vibrational modes of OH (3364 and 3348 cm^−1^). The intensities of these bands grow concurrently with the increase in xylitol concentration. The remaining unmarked bands are related to the different polymers used. By selecting the most intense bands (2953 and 3000 cm^−1^), it was possible to monitor the presence and distribution of xylitol within the scaffolds.

The Raman mapping images are displayed above the spectra of the various composite scaffolds. The base polymers are labeled in green, while xylitol is marked in red. In all three polymer types, the control samples are predominantly green, indicating the absence of additives. Upon the addition of xylitol, red signals appear, increasingly with the rise in xylitol concentration, demonstrating its successful incorporation within the fibers. Notably, yellow areas become more evident, particularly in CA-X2, CA-X3, and for all the PLLA composites, suggesting spatial overlaps between the polymer and xylitol. These yellow regions reflect co-localization, indicating strong interaction and good distribution of xylitol within the polymer matrix. This integration becomes more pronounced at higher xylitol contents, confirming the effective dispersion of the filler with the polymer scaffolds.

### 3.3. Mechanical Tests of the Composites to Evaluate Xylitol Incorporation

**Figure 5:**
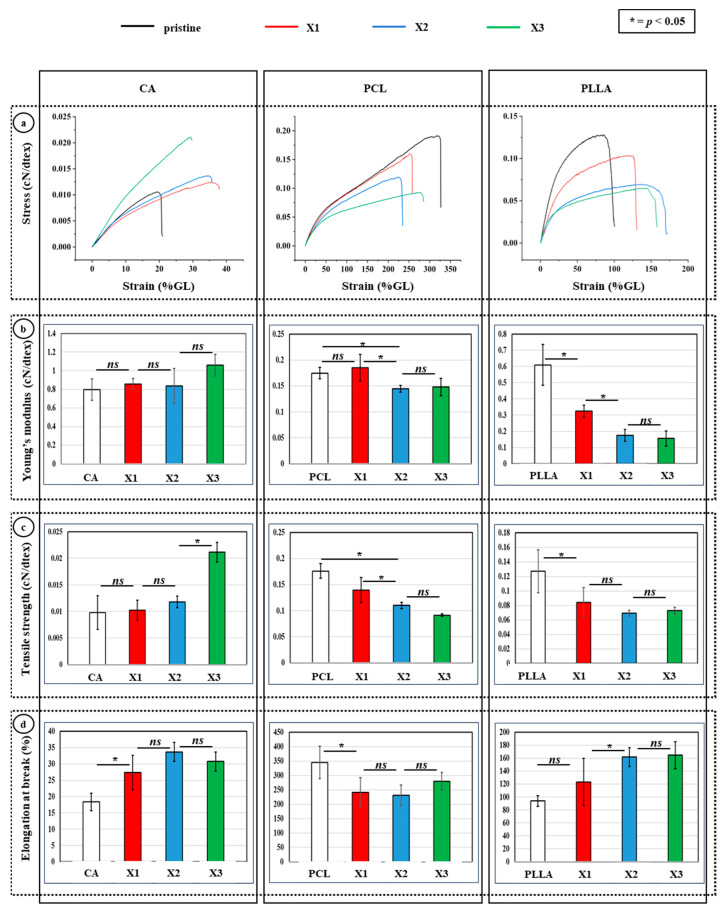
(**a**) Representative stress–strain curves for the different samples, as obtained from tensile testing, with main parameters extrapolated from the tensile-testing experiments: (**b**) Young’s modulus, (**c**) tensile strength, and (**d**) elongation at break (*ns* = non-significant; * = *p* < 0.05).

The mechanical characterization of CA, PCL, and PLLA composites incorporating increasing concentrations of xylitol demonstrated material-specific trends in response to xylitol content. Stress–strain analysis revealed that CA composites exhibited progressively increased strain capacities with rising xylitol content, with CA-X3 displaying the highest deformation before failure (Figure 5a, left panel). Correspondingly, Young’s modulus slightly increased only in CA-X3, even if insignificantly, while CA-X1 and CA-X2 remained statistically similar to pure CA, suggesting that higher xylitol levels enhance stiffness (Figure 5b, left panel). Tensile strength also improved significantly in CA-X3 (Figure 5c, left panel), while elongation at break increased markedly at CA-X1 and remained elevated, indicating enhanced flexibility and mechanical performance at higher xylitol concentrations (Figure 5d, left panel).

In contrast, PCL composites showed a consistent decline in mechanical properties with increasing xylitol. The stress–strain curves (Figure 5a, middle panel) indicated reduced stress tolerance and strain capacity, with significant decreases in both Young’s modulus (Figure 5b, middle panel) and tensile strength (Figure 5c, middle panel) by PCL-X1, and no further significant differences thereafter. Elongation at break (Figure 5d, middle panel) also declined sharply upon initial xylitol incorporation, reflecting compromised ductility and stiffness, likely due to disruption of PCL’s densely packed structure. PLLA-based composites presented an intermediate response. The incorporation of xylitol led to a broader and more diffuse porosity profile and a reduction in both stiffness and strength. Young’s modulus (Figure 5b, right panel) significantly decreased in PLLA-X1, with no significant changes at higher concentrations. Tensile strength (Figure 5c, right panel) was significantly reduced at PLLA-X1 and remained lower across all xylitol-containing PLLA composites. Interestingly, elongation at break (Figure 5d, right panel) increased significantly at PLLA-X2 and PLLA-X3, suggesting enhanced ductility, potentially due to increased porosity or phase separation effects.

### 3.4. DSC Analysis

**Figure 6:**
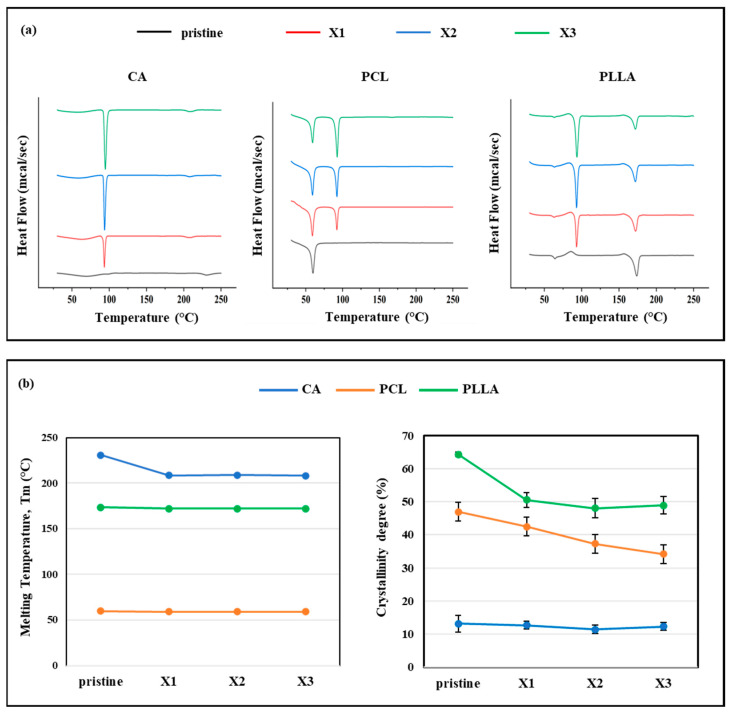
Representative (**a**) DSC curves of the three group of composites and (**b**) extrapolated melting temperature. Degree of crystallinity calculated by the enthalpy obtained from each DSC curve.

The thermal properties of the pristine polymers and their xylitol-based composites were assessed using DSC, with results summarized in Figure 6. The thermograms of the pristine polymers exhibited single endothermic peaks corresponding to their respective melting transitions. Specifically, CA showed a melting temperature peak (T_m_) of 230.6 ± 0.7 °C, PCL melted at 59.8 ± 0.5 °C, and PLLA at 173.6 ± 0.2 °C. In contrast, all xylitol-containing composites displayed an additional pronounced endothermic peak around 100 °C, which was absent in the thermograms of the pristine polymers. This peak is attributed to the melting of free or loosely bound xylitol within the polymer matrices, confirming successful incorporation of the additive. The inclusion of xylitol resulted in a slight reduction in the melting temperature of each polymer matrix. For instance, in CA composites, T_m_ decreased to 208.5 ± 0.4 °C in CA-X1 and to 208.0 ± 1.0 °C in CA-X3, compared to the pristine CA (Figure 6b, left panel). Similarly, the T_m_ of PLLA composites dropped slightly to 172.0 ± 0.2 °C, and in PCL composites to 59.1 ± 0.3 °C, indicating a modest plasticizing effect of xylitol on thermal transitions. Concurrently, the intensity of the main melting peak in PLLA and PCL composites slightly diminishes, suggesting possible partial miscibility or phase separation between polymer and additive (Figure 6b, left panel). A more pronounced effect was observed on the degree of crystallinity (%C), which decreased progressively with increasing xylitol content. In PCL samples, crystallinity dropped from 47.0 ± 4.1% in the pristine polymer to 34.1 ± 1.5% in PC-X3 (Figure 6b, right panel). PLLA exhibited a similar trend, with %C decreasing from 64.3 ± 0.7% in the pristine form to 48.9 ± 2.7% in PL-X3. In CA, which is inherently semicrystalline, a slight crystallinity reduction was reported from 13.1 ± 2.5% to 11.4 ± 1.3% in CA-X2 and up to 12.3 ± 1.1% in CA-X3, indicating how values did not significantly change across all the formulations (Figure 6b, right panel).

### 3.5. In Vitro Bacterial Testing

**Figure 7:**
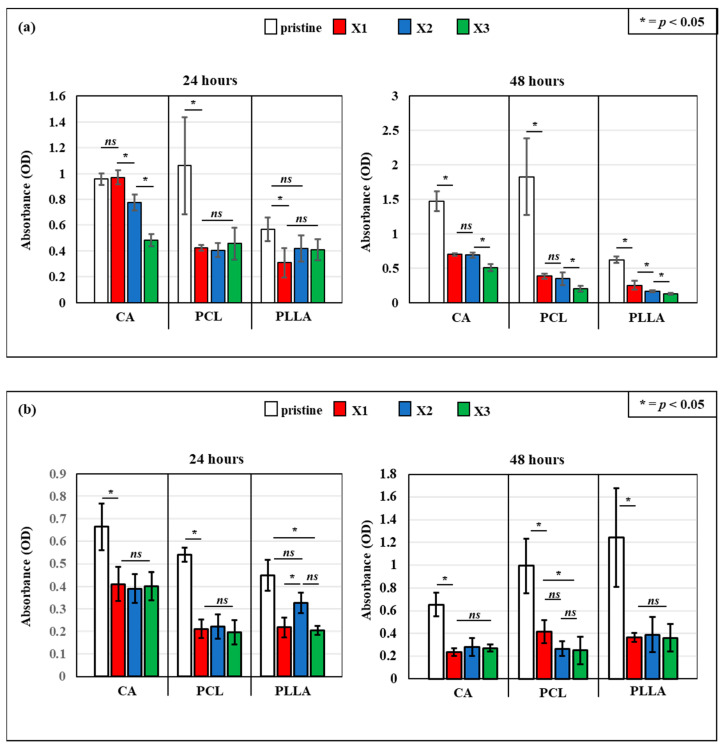
WST adsorption after 24 and 48 h of testing with *E. coli* (**a**) and *S. aureus* (**b**) on the different composites, as a function of the amount of xylitol (*ns* = non-significant; * = *p* < 0.05).

After undergoing blending with increasing concentrations of xylitol (X1, X2, X3), the antibacterial activity of the electrospun scaffolds based on cellulose acetate (CA), polycaprolactone (PCL), and poly(lactic acid) (PLLA) was evaluated using the WST assay after 24 and 48 h of exposure to *Escherichia coli* and *Staphylococcus aureus*, and the results are displayed in Figure 7.

At 24 h (Figure 7a, left panel), all pure polymer samples without xylitol showed high *E. coli* viability, indicated by higher absorbance values. However, for CA and PCL, the incorporation of xylitol led to a clear concentration-dependent reduction in bacterial viability across all polymer types. The reduction was especially marked at higher concentrations of xylitol (X2 and X3), with CA–X3 and PCL–X3 showing the most significant drops. Between the three pristine samples, PLLA displayed the lowest bacterial activity. The xylitol addition enhanced antibacterial performance but not significantly as in the case of CA and PCL composites. After 48 h (Figure 7a, right panel), a similar trend was observed, but, especially for PCL and PLLA, the antibacterial effect appeared more pronounced overtime. For PCL in particular, the viability decreased by ~80%, while in PLLA, the viability was reduced by ~50%. The bacterial viability on all xylitol-containing composites remained lower than the controls and, for PCL and PLLA composites, the bacterial viability reduced overtime. Also, for CA-X1 and CA-X2, there was a clear effect, while CA-X3, despite presenting the lowest OD value, remained constant overtime. Notably, PCL and PLLA matrices containing the highest amount of xylitol (PCL-X3 and PLLA-X3) resulted in the lowest absorbance values, indicating substantial inhibition of bacterial proliferation.

The WST assay results by electrospun scaffolds composed of CA, PLLA, and PCL treated against *Staphylococcus aureus* when loaded with increasing concentrations of xylitol are reported in Figure 7b. At both 24 and 48 h, pristine polymers showed relatively high bacterial viability, particularly in the case of CA. However, the incorporation of xylitol led to a marked and concentration-dependent reduction in metabolic activity across all polymer types. At 24 h (Figure 7b, left panel), CA-based composites showed a sharp initial decrease in viability even at the lowest xylitol concentration (X1), but no difference was observed between the three CA composite. The same trend was reported for PCL samples, while for PLLA composite, the PLLA-X2 did not show statistical difference with the pristine sample, despite a slight decrease in absorbance. Contrastingly, PCL-X1 and PCL-X3 demonstrated an antimicrobial effect. At 48 h, between all the composites, CA samples exhibited a more gradual decline, with the highest concentration (X3) showing the strongest antibacterial effect (Figure 7b, right panel). PCL composites, which already had moderate antibacterial performance, displayed low bacterial viability with xylitol addition, especially at X2 and X3. PLLA composites showed a slight increase in viability compared with the one observed at 48 h but still significantly lower than the pristine one. These findings confirm that xylitol effectively enhances the bacteriostatic properties of electrospun CA, PLLA, and PCL scaffolds, making them highly suitable for biomedical applications such as wound dressings, antimicrobial barriers, and infection-preventive materials.

**Figure 8:**
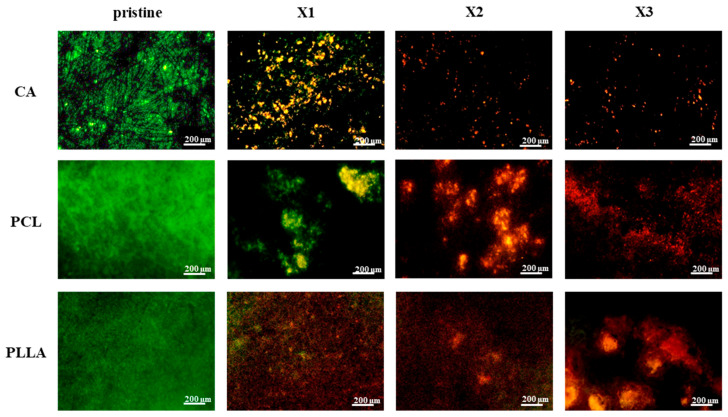
Dead/live staining after 48 h of testing with *E. coli* on the different composites of CA, PCL and PLLA with increasing concentration of xylitol (from X1 (lower) to X3 (higher)). Living bacteria are stained with CFDA and labeled in green, while dead cells are stained with PI and marked in red.

The results of the live/dead staining procedure using CFDA/PI of *E. coli* cultured for 48 h are reported in Figure 8. Pristine CA, PCL, and PLLA displayed widespread green fluorescence, indicating substantial live bacteria and biofilm formation, thus lacking effective *E. coli* growth inhibition. However, xylitol incorporation into the polymer matrices clearly impacted bacterial viability. Across all three polymers, increasing xylitol concentration (X1 to X3) correlated with reduced green (live) and increased red (dead) fluorescence.

**Figure 9:**
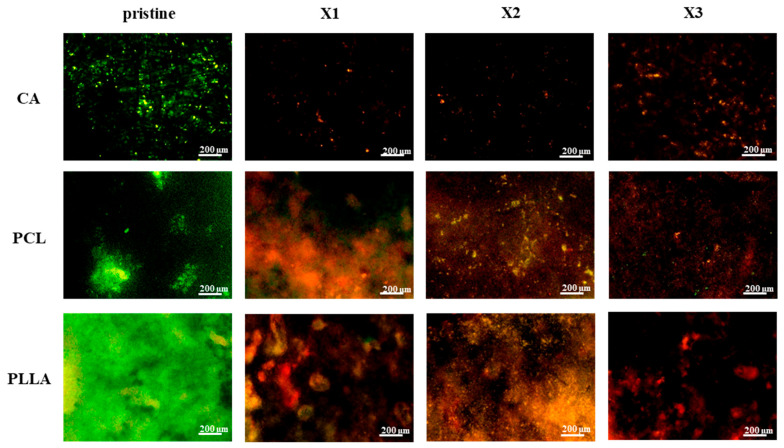
Dead/live staining after 48 h of testing with *S. aureus* on the different composites of CA, PCL and PLLA with increasing concentration of xylitol (from X1 (lower) to X3 (higher)). Living bacteria are stained with CFDA and labeled in green, while dead cells are stained with PI and marked in red.

This effect was most prominent in X2 and X3 composites, where dominant red signals indicated significantly higher *E. coli* mortality, suggesting a concentration-dependent antibacterial effect of xylitol-modified CA, PCL, and PLLA fibrous scaffolds. Figure 9 presents live/dead staining of *S. aureus* after 48 h on pristine and xylitol-containing composite fibers. Pristine CA, PCL, and PLLA exhibited substantial green fluorescence, indicating significant live *S. aureus* populations and no inherent growth prevention. Conversely, xylitol incorporation led to increased antibacterial activity against *S. aureus* with rising concentrations.

CA composites at even the lowest xylitol concentration (X1) showed near-absent green fluorescence and few red signals, revealing strong inhibition. PCL composites transitioned from predominantly green to mixed green/red in X1, then to dominant red and reduced green in X2 and X3, indicating concentration-dependent killing. PLLA composites followed a similar trend of decreasing live (green) and increasing dead (red) bacteria with higher xylitol concentrations. Overall, xylitol incorporation conferred significant antibacterial properties against *S. aureus* in all three polymers, generally improving with concentration. Notably, CA composites displayed the most potent effect against *S. aureus* even at the lowest xylitol concentration.

## 4. Discussion

This study investigated the impact of incorporating xylitol, a natural sugar alcohol known for its antibacterial properties, into electrospun fibrous scaffolds composed of cellulose acetate (CA), polycaprolactone (PCL), and polylactic acid (PLLA). A comprehensive characterization of the resulting composite scaffolds was performed, evaluating their morphology, mechanical, chemical, thermal, and antibacterial properties.

### 4.1. Xylitol Content Strongly Affects the Composite Structures and Performances

The contrasting effects of xylitol incorporation on fiber morphology in electrospun CA, PCL, and PLLA scaffolds could be linked to differences in solvent compatibility, polymer–xylitol interactions, and overall solution behavior during electrospinning. In the case of cellulose acetate (CA) dissolved in acetone, xylitol introduced from a water solution not only results in a decreased fiber diameter but also in reduced porosity and increased fiber packing density. This outcome is primarily due to the miscibility of water and acetone, which enables homogeneous integration of xylitol into the CA matrix. The CA fiber diameter could vary depending on the amount of water mixed with the polymer solution; this has been observed by Han et al., who demonstrated that the average diameters of the CA nanofibers could be controlled by changing the composition of the mixed solvent, influencing solution properties as viscosity and conductivity [46]. A possible explanation of the results could be found in the presence of sugar alcohol dissolved in water; due its hydrophilic nature, it could have facilitated hydrogen bonding of CA, leading to improved chain rearrangement and increased solution conductivity. These conditions could have promoted finer jet stretching during electrospinning and tighter fiber deposition, resulting in a denser mat with less inter-fiber spacing.

In contrast, when xylitol is added to PCL or PLLA solutions, dissolved in a chloroform/acetone (3:1) mixture or chloroform, respectively, the behavior diverges significantly. Due to the immiscibility or only partial miscibility of chloroform with water, xylitol cannot properly dissolve into the polymer solution and instead forms a separate phase. This poor dispersion could lead to emulsion-like systems with increased viscosity, micro-aggregation, and reduced electrospinning stability. As a result, the electrospinning jet undergoes less stretching, producing thicker fibers with greater variability. The disrupted solution homogeneity and incomplete integration of xylitol also cause fibers to deposit more loosely, increasing scaffold porosity and slightly reducing fiber density. Moreover, the absence of favorable interactions, since both PCL and PLLA are hydrophobic and lack affinity for xylitol, prevents the kind of molecular-level integration seen with CA, leading to more open, less compact fiber structures. Another important feature that characterizes all the three types of polymers is the presence of beads at the highest presence of xylitol. This could be attributed to several interconnected factors related to the changes in the electrospinning solution properties, especially surface tension, conductivity, and viscosity. Adding xylitol could increase the overall surface tension of the polymer solution, making it more difficult for the electrospinning jet to elongate uniformly, favoring the formation of droplets, which then solidify as beads along the fibers or as discrete beads. Another cause could be represented by high conductivity: high amount of xylitol, which is a polar molecule, could have increased the electrical conductivity of the electrospinning solution, leading to an increased charge density on the jet. This latter could have caused stronger Coulombic forces that disrupted the stable elongation of the jet, leading to whipping instabilities and formation of beads.

The contact angle analysis highlights the pivotal role of xylitol in modulating the surface wettability of electrospun scaffolds. Across all polymer systems, CA, PCL, and PLLA, the addition of xylitol consistently enhanced surface hydrophilicity, reflecting its strong affinity for water and high polarity [47,48]. This shift in wettability can be attributed to the presence of hydroxyl groups in xylitol, which likely migrate toward the fiber surface or become exposed during fiber formation, thereby reducing surface tension. Notably, the degree of wettability improvement varied among the polymers, suggesting that the interaction between xylitol and the polymer matrix is influenced by their intrinsic chemical compatibility and by the high amount of -OH groups presented in the xylitol structure. In particular, a high pronounced hydrophilicity observed in CA- and PLLA-based composites may result from better miscibility and hydrogen-bonding potential between xylitol and the polymer chains. In contrast, PCL, being more hydrophobic, exhibited limited interaction with xylitol, leading to less variation. Evident changes in surface properties were observed only at higher concentration of xylitol where the molecule expressed an increased surface wettability, reducing the polymer hydrophobicity. These findings underscore xylitol’s potential not only as a bioactive additive but also as a surface modifier, with implications for improving scaffold performance in applications where cell attachment, or moisture interaction are influenced by surface hydrophilicity.

### 4.2. Xylitol Decreased Crystallinity, and Influenced Mechanical Properties

The mechanical response of xylitol-incorporated electrospun scaffolds exhibited distinct trends depending on the polymer matrix, highlighting the influence of polymer–filler compatibility. In CA-based composites, mechanical performance improved with increasing xylitol content, as shown by the progressive rise in elongation at break and tensile strength. This enhancement suggests that xylitol acts as a plasticizing agent within the CA network, potentially forming hydrogen bonds that improve chain mobility and cohesion without significantly compromising stiffness. The slight, though non-significant, increase in Young’s modulus at the highest xylitol concentration further supports the idea of structural reinforcement through favorable molecular interactions. Previous tests conducted on CA but using another polyol like the glycerol, showed how the incorporation of the plasticizer influenced the mechanical and thermal properties of the CA scaffolds, also increasing the crystallinity; these improvements were described as a result of a rearrangement of the chains of the polymer matrix and occurred via hydrogen bonds between glycerol and CA [49]. Conversely, PCL composites experienced a notable decline in mechanical properties upon xylitol addition. The sharp drop in tensile strength, Young’s modulus, and elongation at break, particularly evident in the initial xylitol-containing formulation, indicates phase separation and poor compatibility. Xylitol likely disrupts PCL’s crystalline packing and forms heterogeneities that compromise mechanical integrity. PLLA exhibited an intermediate behavior: while stiffness and strength were reduced, particularly at lower xylitol levels, elongation at break improved at higher concentrations, pointing to a dual effect of structural disruption and increased ductility. This may be attributed to broader pore distribution and partial miscibility, resulting in softer, more deformable scaffolds. The thermal analysis of the scaffolds further supports the trends observed in mechanical behavior and reveals important insights into the phase interactions between xylitol and the polymers. The appearance of a distinct endothermic peak around 100 °C in all xylitol-containing composites confirms the presence of free or loosely bound xylitol and verifies its successful incorporation into the electrospun fibers. Additionally, the modest decrease in melting temperature across CA, PCL, and PLLA suggests a plasticizing effect of xylitol, which likely disrupts the regularity of polymer chain packing. This is particularly evident in the degree of crystallinity, which dropped significantly in PCL and PLLA composites with increasing xylitol content, indicating reduced structural order and the presence of amorphous regions due to limited miscibility. These findings are confirmed by the results from other published works that used other sugar alcohols instead like glycerol, demonstrating how its incorporation in PCL progressively disrupted the crystallinity and reduced the melting temperature due to branching and consequently enhancing its water absorption capacity [50]. Even PLA composite scaffolds that were modified with the addition of glycerol exhibited the same performance and variations in mechanical properties and crystallinity that were observed with the addition of xylitol [51]. A crescent concentration of glycerol decreased the tensile strength of PLA composites and consequently increased the elongation at break. In another study, reported by Lv et al., where glycerol was mixed with PLA to evaluate its effect as plasticizer, the crystallinity degree decreased and affected the thermal and mechanical properties [52]. The reduced crystallinity in PCL and PLLA not only contributes to their mechanical softening but also reflects a lack of cohesive integration of xylitol within the polymer matrix. In contrast, CA, which is inherently less crystalline, showed minimal changes in crystallinity, aligning with its greater compatibility with xylitol.

### 4.3. Significant and Stable Antibacterial Effect Provided by the Xylitol Against S. aureus and E. coli over Time

The integration of xylitol into electrospun CA, PCL, and PLLA scaffolds notably enhanced their antibacterial performance against both *Escherichia coli* and *Staphylococcus aureus*, with the effects varying depending on polymer type, bacterial species, and exposure time. Pristine scaffolds exhibited negligible antibacterial activity, underscoring the necessity of functional additives like xylitol. The bacteriostatic mechanism of xylitol, primarily driven by metabolic interference and anti-adherence effects, manifested differently across the two bacterial strains. Against *S. aureus*, a Gram-positive bacterium, the scaffolds exhibited rapid and significant inhibition, even at the lowest xylitol concentrations. This suggests a high susceptibility of *S. aureus* to xylitol released from the polymer matrixes, likely due to enhanced interactions with bacterial surface proteins and disruption of extracellular polysaccharide formation, which are key to adhesion and biofilm development. This has been confirmed by in vitro experiments related to the study of xylitol against the bacteria for skin-related application [41,53,54]. The sugar has the capacity to stop the growth of the colonies by inhibiting the formation of glycocalyx that, along with the fibrin fibers, are responsible in biofilm biomass formation [53]. Furthermore, another work published by Ferreira et al. describes how xylitol also presents anti-adherence properties to function against *S. aureus* [55].

Regarding the *E. coli* testing, the composites showed a gradual and concentration-dependent reduction in viability, especially in CA- and PCL-based composites, as demonstrated by WST assays and live/dead staining. While the antibacterial effect was less immediate, the scaffolds maintained a sustained inhibitory action over 48 h, indicating either slow diffusion or the prolonged effect of xylitol within the fibrous matrix. This suggested that the antibacterial effect of xylitol against *E. coli* is different and more dose-related compared to the effect observed against *S. aureus*. And these findings align with the context-dependent antibacterial efficacy of xylitol reported in the literature; an in vitro study performed by da Silva et al. suggested that xylitol’s impact on *E. coli* may not be substantial unless high concentrations are used, and furthermore, they demonstrated how the sugar presented stronger anti-adhesion properties when compared to the capacity to inhibit the bacterial growth and colony formation [56].

### 4.4. Use and Limitations of Xylitol-Based Scaffolds

The integration of xylitol into electrospun CA, PCL, and PLLA scaffolds introduces a set of characteristics that distinguish these composites from conventional polymer-based bioactive scaffolds. Conventional electrospun polyesters, such as PLLA and PCL, are widely employed for tissue engineering due to their processability and mechanical stability; however, they typically exhibit high stiffness (Young’s modulus in the GPa range) and very slow degradation rates that can extend over several years, often leading to poor compliance with soft tissues and long-term persistence in vivo. By contrast, xylitol-containing scaffolds display mechanical softening and tunable degradation, placing their behavior closer to sugar-derived elastomers and PEG–citrate hybrid systems, which are known for their elasticity and faster resorption [57,58]. This tunability arises from the disruption of crystalline packing and hydrogen bonding induced by xylitol, as confirmed by the reduction in melting temperatures and crystallinity observed in our DSC analysis (Section 4.2, Figure 6). Such thermal and mechanical signatures are in line with the effects reported for glycerol-modified PCL, PVA and PGA systems, where the incorporation of polyols decreased crystallinity and stiffness while increasing ductility and hydrophilicity [50,59,60].

In terms of bioactivity, xylitol offers advantages that differ from more traditional strategies such as the incorporation of bioactive fillers (e.g., hydroxyapatite, bioglass) or chemical functionalization of polyesters. While bioactive fillers can endow scaffolds with osteoconductive or antibacterial properties, they often introduce particle aggregation, inhomogeneous dispersion, and batch-to-batch variability, which can compromise mechanical performance and reproducibility [61,62]. The presence of filler/matrix interfaces also often leads to a deterioration of mechanical properties, which in turn limits the maximum feasible filler content [63]. In our study, Raman mapping (Figure 4) revealed the presence of xylitol-rich domains, indicating some local heterogeneity. However, the overall Raman signal intensity was comparable between batches and patches, confirming a reproducible incorporation of xylitol throughout the scaffold volume. This contrasts with filler-based systems, where inhomogeneity frequently leads to unpredictable mechanical behavior.

PEG-based and citrate elastomer scaffolds typically rely on covalent functionalization or surface coatings to achieve bioactivity, often requiring multi-step synthesis. By comparison, xylitol acts as a single-component bioactive additive, conferring hydrophilicity, metabolic compatibility, and antibacterial activity without additional surface treatments. The stable antibacterial effect demonstrated against both *S. aureus* and *E. coli* (Section 4.3) highlights an intrinsic functionality not typically observed in pristine PLLA, PCL, or CA scaffolds.

Despite these advantages, certain limitations must be acknowledged. The presence of xylitol-rich domains may create local variations in mechanical properties and degradation rates, particularly in hydrophobic matrices such as PCL and PLLA, where miscibility is limited. This microscale heterogeneity could affect uniformity in vivo. Additionally, the current study provides semi-quantitative insights into xylitol content and distribution via Raman and DSC, but absolute quantification (e.g., by HPLC-RI or TGA) will be essential to fully elucidate structure–property relationships. Finally, while the observed antibacterial activity and tunable mechanical properties are promising, long-term in vivo evaluations are needed to determine how these features translate into tissue integration, degradation behavior, and functional performance in physiological environments.

### 4.5. Notes on the Stability of Xylitol in Physiological Environments

Previous studies have shown that xylitol-based polymers and elastomers exhibit good biocompatibility and undergo gradual hydrolytic degradation under physiological conditions, resulting in a controlled release of xylitol without causing adverse tissue reactions. For instance, Bruggeman et al. reported that biodegradable xylitol-based elastomers degrade predictably in vivo, releasing xylitol in a sustained manner while maintaining biocompatibility and structural integrity during the early stages of implantation [64,65]. Additionally, Arruda et al. reviewed xylitol’s physicochemical stability in aqueous and physiological media, emphasizing its high stability, low reactivity, and suitability for biomedical formulations, including controlled release systems [66].

In our study, the polymer matrices are expected to provide a similar gradual diffusion of xylitol in aqueous physiological environments (e.g., PBS, pH 7.4), enabling prolonged antimicrobial activity without abrupt leaching. While detailed quantitative release profiles are beyond the scope of this work, these previous findings strongly support the stability and controlled release behavior of xylitol, which is crucial for potential biomedical applications of our electrospun scaffolds. We are currently planning follow-up studies to systematically evaluate release kinetics and stability under physiological conditions.

## 5. Conclusions

In conclusion, this study demonstrates that the incorporation of xylitol into electrospun CA, PCL, and PLLA scaffolds leads to profound modifications of their physicochemical, mechanical, and biological properties, underscoring the strong influence of polymer–filler interactions. Morphological analyses revealed that xylitol reduced fiber diameter and porosity in CA scaffolds, while inducing larger and more heterogeneous fibers in PCL and PLLA, reflecting differences in solvent–polymer–xylitol interactions and matrix compatibility. These structural changes directly influenced mechanical behavior: CA benefited from improved tensile strength and flexibility at higher xylitol concentrations, whereas PCL composites consistently lost mechanical performance, and PLLA displayed reduced stiffness but a marked increase in ductility. These trends were corroborated by thermal analysis, which showed a reduction in melting temperature and crystallinity, particularly in PCL and PLLA, indicating a plasticizing effect of xylitol on the polymer matrices. Additionally, all three polymers exhibited enhanced surface hydrophilicity with increasing xylitol content, a property that may favor cell adhesion and integration in biomedical settings.

The most significant functional outcome of xylitol incorporation was the strong antibacterial activity imparted to all scaffolds. Both *S. aureus* and *E. coli* growth were inhibited, with *S. aureus* showing rapid and pronounced susceptibility, while *E. coli* inhibition occurred more gradually and was concentration dependent. These results highlight xylitol’s role as a broad-spectrum antibacterial agent capable of enhancing the intrinsic bioactivity of electrospun scaffolds.

Beyond the specific systems studied, these findings suggest that the strategy of incorporating xylitol into electrospun matrices can be generalized to a wider range of polymers. Electrospinning is inherently adaptable to multiple natural and synthetic polymers, and the ability of xylitol to simultaneously modulate morphology, mechanics, wettability, and antibacterial function positions it as a broadly applicable additive. Thus, this approach has strong potential for translation into diverse application areas, including wound dressings, tissue scaffolds, protective filtration systems, and antimicrobial packaging.

Future research should expand this framework by investigating compatibility with other polymer systems, optimizing xylitol distribution to balance mechanical and antibacterial performance, and evaluating long-term stability and in vivo functionality. Taken together, our results position xylitol-modified electrospun fibers as a versatile, low-cost, and scalable platform technology for the development of next-generation functional biomaterials.

## Data Availability

The original contributions presented in the study are included in the article, further inquiries can be directed to the corresponding author.
